# Mechanized wet direct seeding for increased rice production efficiency and reduced carbon footprint

**DOI:** 10.1007/s11119-024-10163-8

**Published:** 2024-07-12

**Authors:** Nguyen Van Hung, Tran Ngoc Thach, Nguyen Ngoc Hoang, Nguyen Cao Quan Binh, Dang Minh Tâm, Tran Tan Hau, Duong Thi Tu Anh, Trinh Quang Khuong, Vo Thi Bich Chi, Truong Thi Kieu Lien, Martin Gummert, Tovohery Rakotoson, Kazuki Saito, Virender Kumar

**Affiliations:** 1 International Rice Research Institute, Los Baños, Laguna, 4031 Philippines; 2 Cuu Long Rice Research Institute, Can-Tho City, Vietnam

**Keywords:** Crop establishment, Sustainable production, Low carbon, Climate change, Agrifood, Resilience

## Abstract

Crop establishment is one of the major rice production operations that strongly affects rice production, productivity, and environmental impacts. This research introduced a new technology and provided scientific evidence for the benefits of mechanized wet direct seeding (mDSR) of rice as compared with the other crop establishment practices commonly applied by farmers for wet direct seeded rice in Mekong River Delta in Vietnam, such as seeding in line using drum-seeder (dDSR) and broadcast seeding (bDSR). The experiment was implemented across two consecutive rice cropping seasons that are Winter-Spring season and Summer-Autumn season in 2020–2021. Treatments included (1–3) mDSR with seeding rates of 30, 50, and 70 kg ha^− 1^, (4) dDSR with 80 kg ha^− 1^ seed rate, and (5) bDSR as current farmer practice with seeding rate of 180 kg ha^− 1^. The fertilizer application was adjusted as per seeding rate with 80:40:30 kg ha^− 1^ N: P_2_O_5_: K_2_O with lower seed rate 30 and 50 kg ha^− 1^ in mDSR; 90:40:30 kg ha^− 1^ N: P_2_O_5_: K_2_O with medium seed rate of 70 to 80 kg ha^− 1^; and 115:55:40 kg ha^− 1^ N: P_2_O_5_: K_2_O with high seed rate of 180 kg ha^− 1^ in bDSR. Mechanized wet direct seeding rice with a lower seed rate of 30 to 70 kg ha^− 1^ and fertilizer rate by 22–30% reduced variation in seedling density by 40–80% and in yield by 0.1 to 0.3 t ha^− 1^ and had similar yield to bDSR. In consequence, N productivity was 27 and 32% higher in mDSR as compared to bDSR during the Winter-Spring season and Summer-Autumn seasons, respectively. The use of lower seed rate and fertilizer in mDSR also led to higher income and lower carbon footprint (GHGe per kg of paddy grains) of rice production than the currently used practices of bDSR. Net income of mDSR was comparable to that of dDSR and higher by 145–220 and 171–248 $US than that of bDSR in Winter-Spring season and Summer-Autumn, respectively. The carbon footprint of mDSR rice production compared to bDSR was lower by 22–25% and 12–20% during the Winter-Spring and Summer-Autumn seasons, respectively. Given the above benefits of farming efficiency, higher income, and low emission, mDSR would be a technology package that strongly supports sustainable rice cultivation transformation for the Mekong River Delta of Vietnam.

**Supplementary Information:**

The online version contains supplementary material available at 10.1007/s11119-024-10163-8.

## Highlights

Mechanized direct seeding decreased the seed rate by 61–83% compared to broadcast seeding.

Mechanized direct seeding increased nitrogen productivity by 27–32% compared to broadcast seeding.

Mechanized direct seeding decreased rice carbon footprint by 19–24% compared to broadcast seeding.

**Supplementary Information:**

The online version contains supplementary material available at 10.1007/s11119-024-10163-8.

## Introduction

Achieving food and nutritional security while reducing the environmental impact of food production and improving rural communities’ well-being in a changing climate are the grand challenges of the 21st century (Foley et al. 2011; Springmann et al. 2018). Globally, 505 million tons of milled rice is produced annually (Statista, 2021), which serves as a staple food for approximately 3.5 billion people; therefore, it is a critical cereal crop to global and regional food security (FAO, 2020).

In Vietnam, rice is considered the priority commodity and it provides livelihoods to two-thirds of the rural labor force. With over 40 million tons of rice production annually, Vietnam is the world’s 5th largest rice producer and second-largest rice exporter (OECD, 2020). Rice production constitutes 30% of the country’s total agricultural production value. About 50% of rice production comes from the Mekong River Delta, which is considered the main rice basket of Vietnam (GSO, 2019). Although rice production is crucial for food security in the country, rice is also one of the major contributors to greenhouse gas (GHG) emissions in Vietnam. It is estimated that paddy production emits about 45 million tons of CO_2_ equivalent – 18% of total national GHG emissions (Tran et al., 2019). Therefore, to ensure both food and environmental security, it is important to maintain or increase productivity while reducing the environmental footprint of rice production including reducing GHG emissions.

Crop establishment is one of the major rice production operations that affects rice production, productivity, and environmental impacts (Kumar & Ladha, 2011; Devkota et al., 2020). In Vietnam, direct wet seeding of rice, including manual broadcasting and blower seeding with a high seed rate (∼ 180 kg ha^-1^) is the most dominant method of rice establishment and is practiced in about 69% of the total rice area. In the Mekong River Delta of Vietnam, direct wet seeding of rice is the preferred method of rice establishment by almost all farmers, except seed producers who use the transplanting method (Nguyen-Van-Hung et al., 2022). Similarly, broadcast direct wet seeding of rice (bDSR) is widely adopted by smallholder farmers in other Southeast Asian countries such as Cambodia, Malaysia, Myanmar, Philippines, and Thailand to overcome the rural drivers of agricultural change including labor, water, and energy scarcity and rising cost of cultivation (Kumar & Ladha, 2011; Farooq et al., 2011; Kaur & Singh, 2017).

Some of the major issues with the current bDSR system in Mekong River Delta, in Vietnam and other southeast Asian countries include – high seed rate, overuse of nitrogenous fertilizer and pesticides, and crop lodging associated with high seed rate and surface broadcast seeding (Stuart et al., 2018; Gummert et al., 2018). Overuse of inputs coupled with the rising cost of these inputs are making smallholder rice production economically and environmentally less sustainable (Tu et al., 2015; Tong, 2017). In addition to economic and environmental impact, high seed rates, N fertilizer rates, and pesticides can lead to favorable conditions for higher insect-pest incidences and crop lodging (Stuart et al., 2014; Wang et al., 2014; Horgan et al., 2021). Higher lodging leads to higher post-harvest losses (Gummert et al., 2018). Reasons for use of high seed rates are that (i) farmers use their own saved seeds which are generally of poor quality (Chhun et al., 2020; Chauhan et al., 2015), and (ii) they avoid risk of non-uniform crop establishment, that requires additional labor for transplanting. Farmers’ own saved seeds also pose a high risk of weed seed contamination and seed-borne diseases (Chhun et al., 2020; Rao et al., 2017). Overall, high agricultural inputs and inefficient practices are major causes of the high carbon footprint (CF) of rice production (Nguyen-Van-Hung et al., 2022; Li et al., 2019).

To address the above-mentioned issues, mechanized and precision DSR has been recently developed and introduced in Asia for both dry and wet seeding with promising advantages such as reducing seed rate, uniform crop establishment, and increasing rice productivity and agronomic and labor use efficiency (Kumar & Ladha, 2011; Yoo et al., 2008; Shenggang et al., 2016; Minghua et al., 2021; Su et al., 2022). Mechanized DSR with a low seeding rate can also enable farmers to use good quality seed. Fertilizer recommendations, especially N rates are also lower for DSR with lower seed rate, hence mechanized seeding offers an opportunity to reduce N rates in rice production (Stuart et al., 2018; Nguyen-Van-Hung et al., 2022; Kaur & Singh, 2017; Ahmed et al., 2015). Other potential benefits associated with mechanized or line seeding with low seed rates include lower risk of crop lodging and pest and disease infestation (Wang et al., 2021; Chaudhary et al., 2022). Furthermore, mechanized DSR combined with efficient water management such as alternate wetting and drying can improve yield, and water productivity, and reduce GHG emissions (Caton et al., 2002; Polthanee et al., 2008; Kumar & Ladha, 2011; Chauhan et al., 2015; Sarangi et al., 2014; Chakraborty et al., 2017; Yoo et al., 2008; Shenggang et al., 2016; Minghua et al., 2021).

Despite many records of studies related to mechanized direct seeding technique for rice in Asia for example Pandey et al. (2002) and Matsubara et al. (2022), no record of studies that involve seed and applied nutrient productivity and C footprint assessments particularly the mechanized direct line seeding for wet-soil rice production in the Mekong River Delta of Vietnam is available. Although Nguyen et al. (2022) tested various mechanized crop establishments and measured GHG emissions in the region, the mechanized direct seeding for wet soil was not included. This study aimed to test the performance of a novel mechanized line seeding technology for wet DSR. The hypothesis is that mechanized line seeding (mDSR) with a lower seed rate and adjusted N rates is better than the current practice of broadcast seeding using a blower (bDSR) with a high seed rate and drum seeding (dDSR). The comparison will be quantified based on the indicators representing crop productivity and sustainability, such as agronomic input use efficiency, plant density and uniformity, grain yield, profitability (net income), and CF (GHGe per kg paddy grains produced) in rice production in Mekong River Delta.

## Materials and methods

All methods included in the research, such as the experimental design, and measurement of seedling uniformity, yield, and sustainable performance indicators, are under the guidelines of the International Rice Research Institute (IRRI) or global standards, which are indicated in the specific sections and parameters below. In addition, the manuscript was internally reviewed and approved by IRRI.

### Site and crop descriptions

**Fig. 1:**
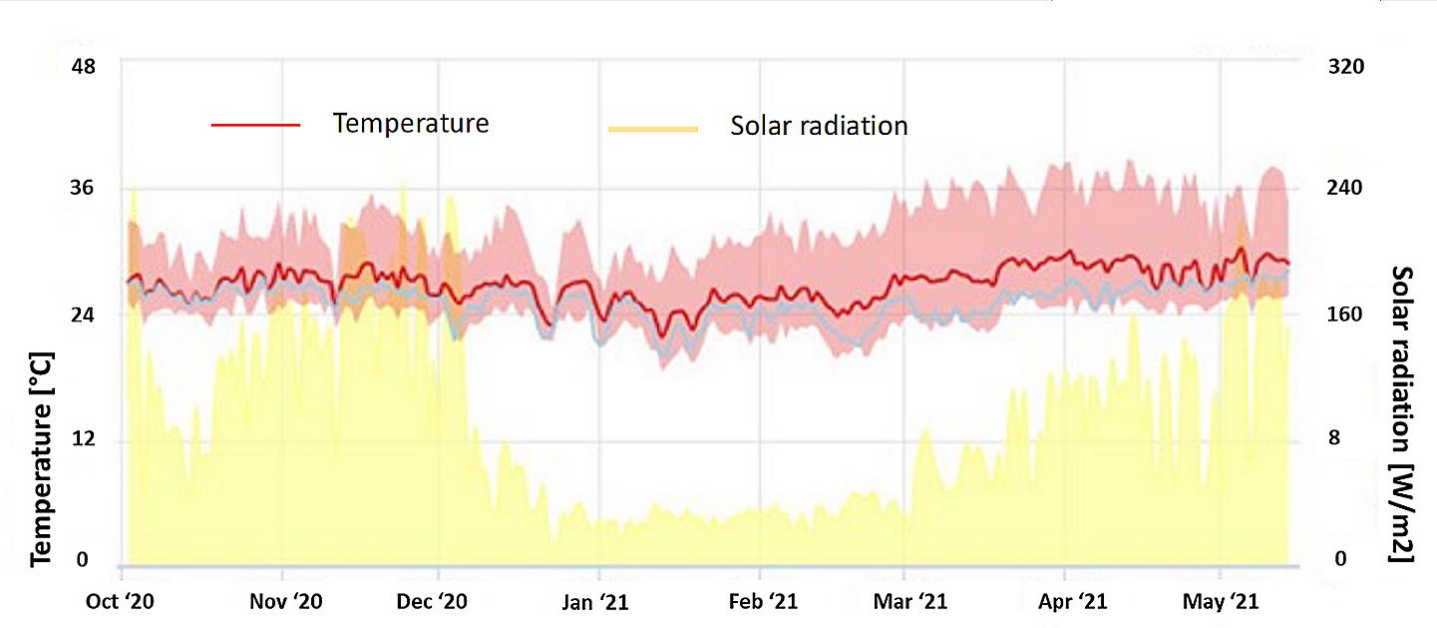
Climate parameters during the experiment

The field study was conducted at the Cuu Long Rice Research Institute (CLRRI) located in Can Tho province of Vietnam (latitude 10.122566, longitude 105.57349) and implemented across two consecutive rice cropping seasons that are Winter-Spring season from 27 November 2020 (seeding) to February 2021 (harvest); and the Summer-Autumn season, or early wet season, from March (seeding) to May 2021 (harvest). Climate parameters during the experiment are shown in Fig. 1. The experiment used the certified seed of the OM18 variety as it is well suitable for the slightly saline soil and ecology and is widely used the MRD of Vietnam. OM18 is a short-duration rice variety with 95–105 days of growth time and plant height of 100–110 cm (Asia Commodity Development, 2021).

Table 1 shows crop calendars of the two experiment seasons, including the major activities such as seed and land preparations, crop establishment, fertilizer and pesticide applications, water management, and harvesting. The seeding date (DAS = 0) of the Winter-Spring and Summer-Autumn seasons were in Nov 2020 and May 2021, respectively. The activities before or after seeding are reflected in (-) or (+), respectively.

**Table 1:** Crop calendar of the two experiment seasons ((-) or (+) = number of days before or after the seeding date)

Activities/ operations	Major features	Crop calendar	
		Winter-Spring 0 = 27/11/2020	Summer-Autumn 0 = 12/05/2021
Soaking seed	Soaking seed in normal water for 36–48 h	-4	-3
Incubating seed	Shade drying for 3 h, then cover with a canvas for 20 h	-1	-1
Land preparation	Using the Kubota L4508VN 4-wheel Tractor (rotavating, puddling, and wet leveling)	-2	-2
Seeding	Following the 5 treatments shown in Table 2	0	0
Fertilizer application	Manual application; of the total fertilizer amount (Table 2):		
• First application	¼ Urea, ½ DAP, and 1/3 Potassium.	10	10
• Second application	½ Urea and ½ DAP	20	20
• Third application	¼ Urea and 2/3 Potassium	44	46
Pesticide application	Using knapsack sprayers;		
• Snail and weed treatments	Molluscicide and Herbicide	-3	-5
• Rice blast, bacteria, and leaf-folder	Insecticide and fungicide	7, 26, 50, 60, 70	7, 10, 22, 32, 40, 48, 62, 73
Water drainages	(drying the fields)	35, 80	35, 75
Yield sampling (crop cut)	Sampling with 5m^2^ /sample for each plot (3 plots/ treatment)	95	95
Harvesting	Kubota DC60 Combine Harvester	98	97
Rice straw	Removal of rice straw from the field (farmer selling straw)	100	100

### Experimental design and crop management

**Fig. 2:**
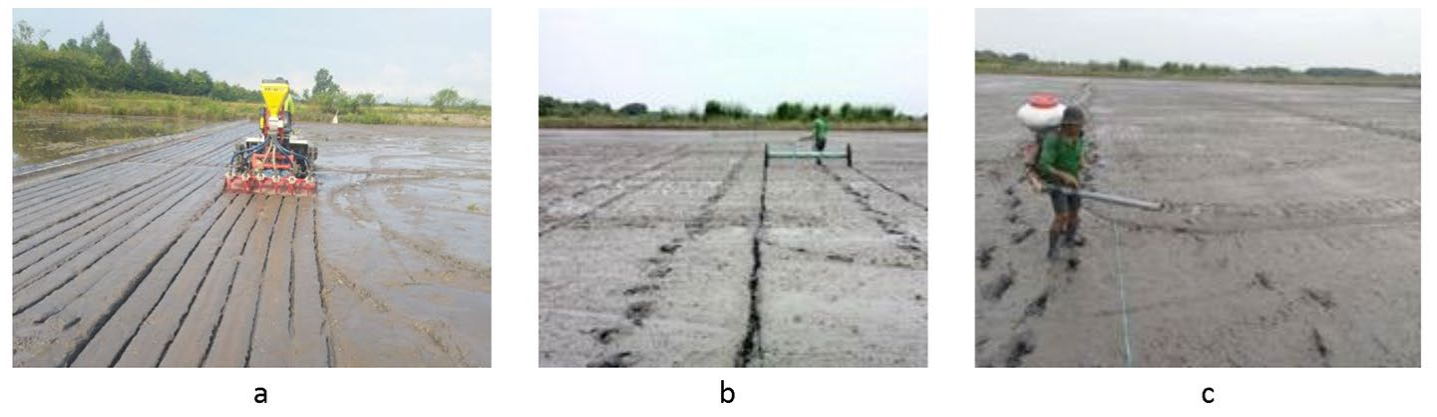
**a.** mDSR seeding (Can Tho City, 27 November 2020). **b.** Drum seeding (Can Tho City, 27 November 2020). **c.** Manual broadcasting (Can Tho City, 27 November 2020)

The experiment was conducted to compare different seeding methods, including mDSR (Fig. 2a), dDSR (Fig. 2b), and bDSR (Fig. 2c). The mDSR scenario was divided into three treatments corresponding to three seeding rates (mDSR-30, mDSR-50, and mDSR-70), while the dDSR and bDSR were considered as the control treatments. The five treatments applied various levels of seed and fertilizer use rates (Table 2). The seeding rates of mDSR were set based on an assumption of at least equal to that of mechanized transplanting in Mekong River Delta of Vietnam (Nguyen-Van-Hung et al., 2022), while the seed rate of dDSR and bDSR were based on the current practices in the area (Stuart et al., 2018; Tho et al., 2021). The fertilizer use rates were adjusted according to the seeding rate based on the practices recommended in the area (Stuart et al., 2018; Nguyen-Van-Hung et al., 2022). A randomized complete block design was applied for the experiment, with three replications distributed into three blocks. The total field area was 1.5 ha distributed to 15 plots (0.1 ha per plot), corresponding to 5 treatments and 3 replications.

**Table 2:** The treatments associated with seed and fertilizer rates

#	Treatment	Methods	Seed rate (kg/ha)	Fertilizers (kg/ha)		
				*N*	*P*_2_O_5_	K_2_O
1	mDSR-30	Mechanized seeding	30	80	40	30
2	mDSR-50		50	80	40	30
3	mDSR-70		70	90	40	30
4	dDSR	Manual drum seeding	80	90	40	30
5	bDSR	Broadcast seeding using a blower	180	115	55	40

### The direct seeding machine

**Fig. 3:**
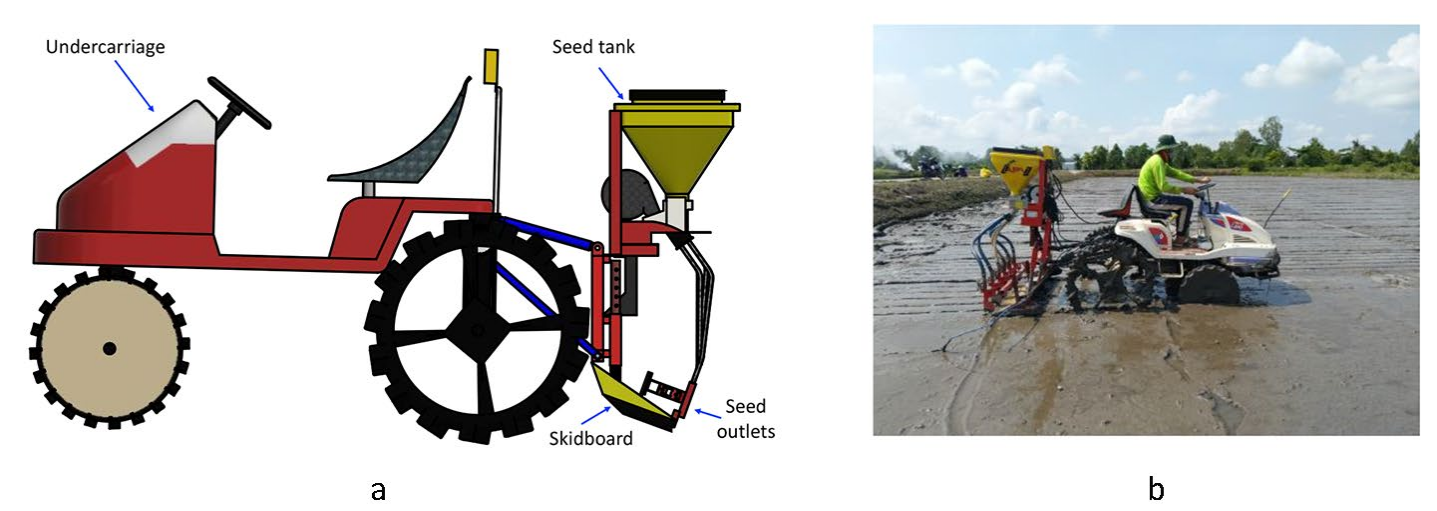
**a.** Schematic diagram of the APV-Austria direct seeder. **b.** The direct seeder operating in the experiment field

The experiment used a direct seeding machine developed by APV-Austria (APV-AT, 2022) (Fig. 3). This machine is a pneumatic seeder with the seeds distributed and conveyed by pressurized air. The machine has six outlets corresponding to 6-line seeding with a working width of 1.2 m. The distance between the consecutive seeded lines is 24 cm.

### Measurement of seedling density

Seedling density was measured about seven days after seeding (DAS), using a quadrat (0.5 m*0.5 m). The samplings were replicated at three positions in each plot. Seedling density was calculated using Equation (Eq.) 1. The standard deviation (SD) was then used to compare the variation in seedling density from the mean across all replicate plots.

1 $$Seedling\,Density=\frac{number\,of\,plants}{quadrat\,sample\,area}\,\,\,\,\,\,\,\,\,\,\,\,\,\,\,(\text{p}\text{l}\text{a}\text{n}\text{t}\text{s}/\text{m}2)$$

### Method of crop cut and yield measurement

We used the crop-cut method to measure grain yield, the same protocol described in Nguyen-Van-Hung et al. (2022). The samples for the crop cut were taken from two 5-m^2^ (2.5 × 2.0 m) quadrants, which were located 5 m from the center of each plot along a cross-diagonal transect. The grain yield was determined at 14% moisture content, calculated based on the fresh paddy weight. The grain samples’ moisture content (MC) was determined using a grinding-type moisture meter (Kett^®^, product code: F511), which was pre-calibrated using the oven method (American Society of Agricultural Engineers, 1982).

### Quantification of partial factor productivity of N, P, and K

Within this study, we analyzed the partial factor productivity of fertilizer, including N productivity (grain_kg N_kg^− 1^), P productivity (grain_kg P_kg^− 1^), and K productivity (grain_kg K_kg^− 1^). The N productivity and P productivity are the performance indicators established by the Sustainable Rice Platform (2019). This calculation approach was also similarly applied to K productivity. These said N, P, and K productivity indicators were calculated based on the amount number of paddy grains produced per kg of N, P, and K applied, respectively.

### *Quantification* of profitability from rice production

Profitability ($US ha^-1^) was calculated by deducting the production cost from the gross income. The production cost accounted for agronomic inputs (seeds, fertilizer, and pesticide) and related labor and service costs (Table 3). In the Mekong River Delta of Vietnam, the service costs such as land preparation, water pumping, crop establishment, fertilizer and pesticide applications, and combine harvesting are paid by farmers based on the field area for the entire crop seasons (Vietnam-currency ha^-1^ season^-1^). Meanwhile, gross income accounted for the freshly harvested grain and rice straw sold at the field. The income from rice straw ($US ha^-1^) was calculated based on the selling price of in-field rice straw ($US t^-1^) and yield of rice straw (t ha^-1^), which was assumed as 60% of paddy grain yield (Nguyen-Van-Hung et al., 2020a). Within this study, we did not include the rental cost of land because it is the same for all treatments and does not affect the comparison. The related cost and price of paddy grains are shown in Table 3.

**Table 3:** Cost of inputs and price of outputs

Inputs		Unit	Value	
			WS	SA
*Agronomic inputs*				
Seeds (OM18)		$US kg^− 1^	0.70	0.70
Urea 46-0-0		$US kg^− 1^	0.33	0.33
DAP 18-46-0		$US kg^− 1^	0.52	0.52
K 0-0-60		$US kg^− 1^	0.37	0.37
NPK 16-16-8		$US kg^− 1^	0.69	0.69
Pesticide for mDSR		$US ha^− 1^	239.13	217.39
Pesticide for dDSR		$US ha^− 1^	282.61	234.78
Pesticide for bDSR		$US ha^− 1^	282.61	260.87
*Operations (crop establishment including seed preparation and reseeding, harvesting included combine harvesting and transporting paddy grains to the bunds)*				
Land preparation (rotavating, pudling, and wet leveling)		$US ha^− 1^	60.87	69.57
Water pumping (irrigation and drainage)		$US ha^− 1^	36.96	47.83
Seeding - bDSR		$US ha^− 1^	69.57	69.57
Seeding – dDSR		$US ha^− 1^	83.48	83.48
Seeding – mDSR		$US ha^− 1^	69.57	69.57
Crop care services (fertilizer and pesticide application)		$US ha^− 1^	413.04	413.04
Harvesting		$US ha^− 1^	130.43	93.48
Price of paddy grains at harvest		$US kg^− 1^	0.34	0.35
In-field rice straw after harvest		$US t^− 1^	5.2	5.2

### Quantification of CF

We used the Life-cycle assessment approach (Nguyen-Van-Hung et al., 2020b) to compute CF (kgCO_2_-eq kg_rice^− 1^). The formulas for quantifying CF were also similarly used in our recent study (Nguyen-Van-Hung et al., 2022), and accounted for the production of agronomic inputs, including seeds and fertilizer (CF_agro−input_), mechanized operations (CF_operation_), soil emissions (CF_soil_), and rice straw management (CF_ricestraw_) (Eq. 2).

2 $$CF = C{F}_{agro-inputs} + C{F}_{operation} + C{F}_{soil} + C{F}_{ricestraw} ({\text{k}\text{g}\text{C}\text{O}}_{2}-\text{e}\text{q} \text{k}\text{g}\_{\text{r}\text{i}\text{c}\text{e}}^{-1})$$

Table 4 shows the CF conversion factors. We used the CF conversion factors for agronomic input production (kgCO2-eq kg^-1^) and fuel consumption in the mechanized operations (kgCO2-eq L^-1^) reported in Ecoinvent (2021) and Nguyen-Van-Hung et al. (2022). The agronomic inputs included seeds, N, P_2_O_5_, and K_2_O used to produce rice. The fuel consumption for mechanized operations included:

- Land preparation applied the same for all treatments: the tractors used diesel of about 21.5 L ha^-1^.
- Crop establishment: the mDSR used diesel of about 0.6 L ha^-1^, the dDSR is operated manually, and the bDSR used gasoline of about 1 L ha^-1^.
- Crop care applied the same for all treatments: the knapsack spraying for all fertilizer and pesticide application used gasoline about 4 L ha^1^.
- Harvesting applied the same for all treatments: the combined harvesting used diesel of about 42.5 L ha^-1^.

We did not consider the CF for water pumping using electric power because it came from a station serving for the a big field area of more than 100 ha. Nevertheless, this CF of water pumping per ha of rice production would not be significant and applied the same for all treatments.

CF_soil_ is calculated based on Eq. 3, accounting for CH_4_ and N_2_O emissions per kg of product. The CH_4_ emission is affected by water management, pre-season soil management, and rice straw incorporation (Sustainable rice platform, 2019), while the N_2_O emission is affected by N use for rice cultivation (IPCC, 2019).

3 $$\begin{array}{l} C{F}_{soil} = (Tim{e}_{grow}*28*E{F}_{default}* S{F}_{water}* S{F}_{pre}*S{F}_{ricestraw}\\+ 265*E{F}_{1FR}*{F}_{fertilizer})/Yield ({\text{k}\text{g}\text{C}\text{O}}_{2}-\text{e}\text{q} \text{k}\text{g}\_{\text{r}\text{i}\text{c}\text{e}}^{-1})\end{array}$$

Where Time_grow_ is the rice-growing period, which was 98 and 97 days for Winter-Spring and Summer-Autumn, respectively (Table 1). The numbers (i.e. 28 and 265) are the Global Warming Potentials of CH_4_ and N_2_O, respectively, for conversion to CO_2_-eq (IPCC, 2013). EF_default_ is the CH_4_ emission factor (kgCO_2_-eq ha^-1^ day^-1^) determined for the Mekong River Delta of Vietnam (Vo et al., 2017). SF_water_ is the scaling factor for water management which accounted for the single drainage scenario (not accounting for the drainage before harvest) as indicated in Table 1. SF_pre_ is the scaling factor for pre-season soil submergence status corresponding to a flood condition lower than 30 days and a non-flood condition lower than 180 days (Sustainable rice platform, 2019). SF_ricestraw_ is the scaling factor for rice straw management. Within this study, rice straw was removed from the field thus, there was no effect of rice straw on CF_soil_. EF_1FR_ is the N_2_O emission factor in flooded rice systems and fertilizer amount of applied N (IPCC, 2019). Yield is the amount of paddy grains harvested and normalized at 14% moisture content on wet basis.

**Table 4:** CF conversion factors

Parameters		CF (GHG emission per unit of inputs)		
		Unit	Value	Sources
Seeds		kgCO_2_-eq kg^− 1^	1.12	a, b,c
Diesel consumption		kgCO_2_-eq L^− 1^	3.58	a, d
Gasoline consumption		kgCO_2_-eq L^− 1^	3.13	a, d
N		kgCO_2_-eq kg^− 1^	5.68	a, b,c
P_2_O_5_		kgCO_2_-eq kg^− 1^	1.09	a, b,c
K_2_O		kgCO_2_-eq kg^− 1^	0.52	a, b,c
Soil emission:				
• EF_default_ of CH_4_ in WS		kgCH_4_ ha^− 1^ day^− 1^	1.7	e
• EF_default_ of CH_4_ in SA		kgCH_4_ ha^− 1^ day^− 1^	2.8	f
• SF_pre_ for pre-season soil management			1	f
• SF_water_ for single drainage			0.71	g
• SF_water_ for multiple drainages			0.55	g
• SF_N_ for Nitrogen use (two drainages during cultivation)		% N applied	0.5	g

*a =* Ecoinvent (2021*), b =* SIMAPRO (2021*), c =* IPCC (2013*), d = Nguyen-Van-Hung et al. (*2022*), e = Vo et al. (*2017), f *=* Sustainable rice platform (2019*), g =* IPCC (2019*).*

### Statistical analysis

Analysis of Variance (ANOVA) was used to evaluate the effects of the contrasting crop establishment-based scenarios on the measured production and environmental parameters using a Least Significant Difference (LSD) at *α = 0.05* to compare the mean values.

## Results

### Seedling density

**Fig. 4:**
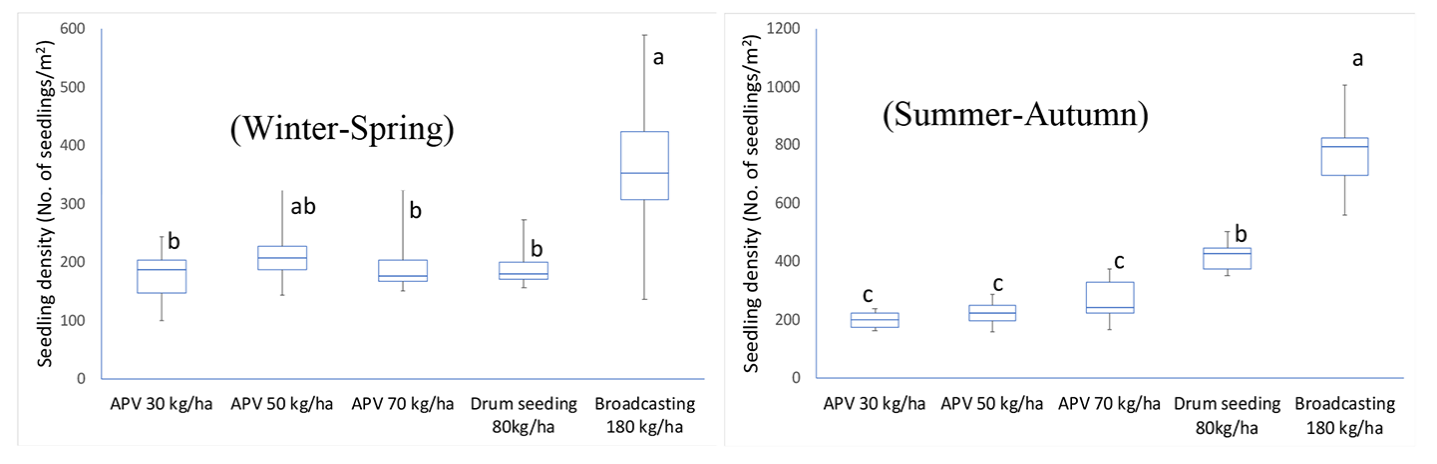
The seedling density of different treatments (No. of seedlings/m^2^) at day seven after seeding. Box plots with the same letters (i.e., **a**, **b**, and **c**) are not significantly different at a 0.05 level of significance following pairwise comparisons using LSD

The seedling density of the bDSR was significantly higher than that of dDSR and mDSR, except in the case of mDSR-50 during the Winter-Spring season (*n* = 3, *p* = 0.05; Fig. 4). On the other hand, compared with dDSR, the seedling density of mDSR was not significantly different during the Winter-Spring season but was lower during the SA season. The seedling density did not differ among mDSR treatments with seeding rates ranging from 30 to 70 kg ha^-1^. During both Winter-Spring and Summer-Autumn seasons, the variation in seedling density (i.e. seedling uniformity) was lower in mDSR and dDSR treatments with line seeding as compared to bDSR. For example, the standard deviation (SD) of mDSR seedlings was lower by 40–65% and 48–80% than that of bDSR in Winter-Spring and Summer-Autumn seasons, respectively.

### Farming efficiency

*Yield*: Table 5 shows the indicators of farming efficiency, including yield, N, P, and K productivity, and net income. During the Winter-Spring season, the average yield was 7.8 t ha^− 1^ and it did not differ among treatments. On the other hand, during the Summer-Autumn season, the yields of mDSR-50, mDSR-70, and dDSR ranged from 3.8 to 3.9 t ha^− 1^ and were 8.6 to 11.0% higher than that of the mDSR-30 and bDSR. However, the yield of mDSR-30 did not differ from bDSR. During the Summer-Autumn season, the yield was much lower than that of Winter-Spring season because of over-raining, low radiation (Fig. 1), and higher insect-pest and disease incidences. In Winter-Spring season, mDSR reduced the yield variability from 0.1 to 0.3 t ha^− 1^ compared to bDSR.

*Partial factor productivity of N, P, and K*: In both seasons, mDSR-30, mDSR-50, mDSR-70, and dDSR generated the highest N, P, and K productivity as a consequence of higher yield and lower fertilizer inputs compared with the bDSR (Table 2). As compared to bDSR, N application was 35 kg ha^− 1^ (30%) lower in mDSR-30 and mDSR-50 and 25 kg ha^− 1^ (22%) lower in mDSR-70 and dDSR).

*Net income*: The differences in agronomic inputs and yields also lead to different net incomes. The mDSR generated a net income of 1,532-1,607 and 184–261 $US ha^− 1^ during Winter-Spring and Summer-Autumn seasons, respectively. This value was comparable to dDSR but US$ 145 to 220 ha^− 1^ (10–16%) and US$ 171 to 248 ha^− 1^ ( (14–21 times) higher than bDSR in Winter-Spring and Summer-Autumn seasons, respectively. These results suggest that line seeding with a lower seed rate using mechanized seeding or drum seeder can enhance farm efficiencies and profitability of the farmers as compared to the currently used practice of broadcast DSR with a high seed rate.

**Table 5:** Farming efficiency indicators of rice production across the five field trial treatments in the Winter-Spring and Summer-Autumn seasons in Can Tho, Vietnam

	mDSR-30		mDSR-50		mDSR-70		dDSR		bDSR	
***Winter-Spring***										
Grain yield 14% (t ha^− 1^)	7.8	(0.3)	7.9	(0.5)	7.7	(0.4)	8.0	(0.3)	7.8	(0.6)
N productivity (grain_kg N_kg^− 1^)	97.0	(3.8)^a^	98.7	(6.0) ^a^	86.0	(4.0) ^b^	89.3	(3.8) ^a^	67.6	(5.6) ^c^
P productivity (grain_kg P_kg^− 1^)	440.7	(17.1) ^a^	448.5	(27.4) ^a^	439.5	(20.7) ^a^	456.7	(19.3) ^a^	321.1	(26.5) ^b^
K productivity (grain_kg K_kg^− 1^)	323.2	(12.5) ^a^	328.9	(20.1) ^a^	322.3	(15.2) ^a^	334.9	(14.1) ^a^	242.8	(20.1) ^b^
Net income (USD ha^− 1^)	1,562		1,607		1,532		1,558		1,387	
**Summer-Autumn**										
Grain yield (t ha^− 1^)	3.5	(0.3) ^b^	3.8	(0.3) ^a^	3.8	(0.4)^a^	3.9	(0.3)^a^	3.5	(0.4) ^b^
N productivity (grain kg N kg^− 1^)	43.2	(4.1) ^a^	47.8	(3.1) ^a^	42.0	(4.1) ^a^	43.3	(3.2) ^a^	30.4	(3.1) ^b^
P productivity (grain kg P kg^− 1^)	196.2	(18.5) ^a^	217.5	(14.2) ^a^	215.0	(21.0) ^a^	221.5	(16.1) ^a^	144.5	(14.6) ^b^
K productivity (grain kg K kg^− 1^)	143.9	(13.6) ^a^	159.5	(10.4) ^a^	157.6	(15.4) ^a^	162.4	(11.8) ^a^	109.3	(11.0) ^b^
Net income (USD ha^− 1^)	184		261		222		236		13	

*Standard errors follow the mean values in parentheses. Within a particular row, numbers followed by different letters are significantly different by the least significant difference at α = 0.05 (n = 3). N, P, and K productivity are the partial factor productivity of N, P, and K, respectively*

### Carbon footprint (CF)

**Fig. 5:**
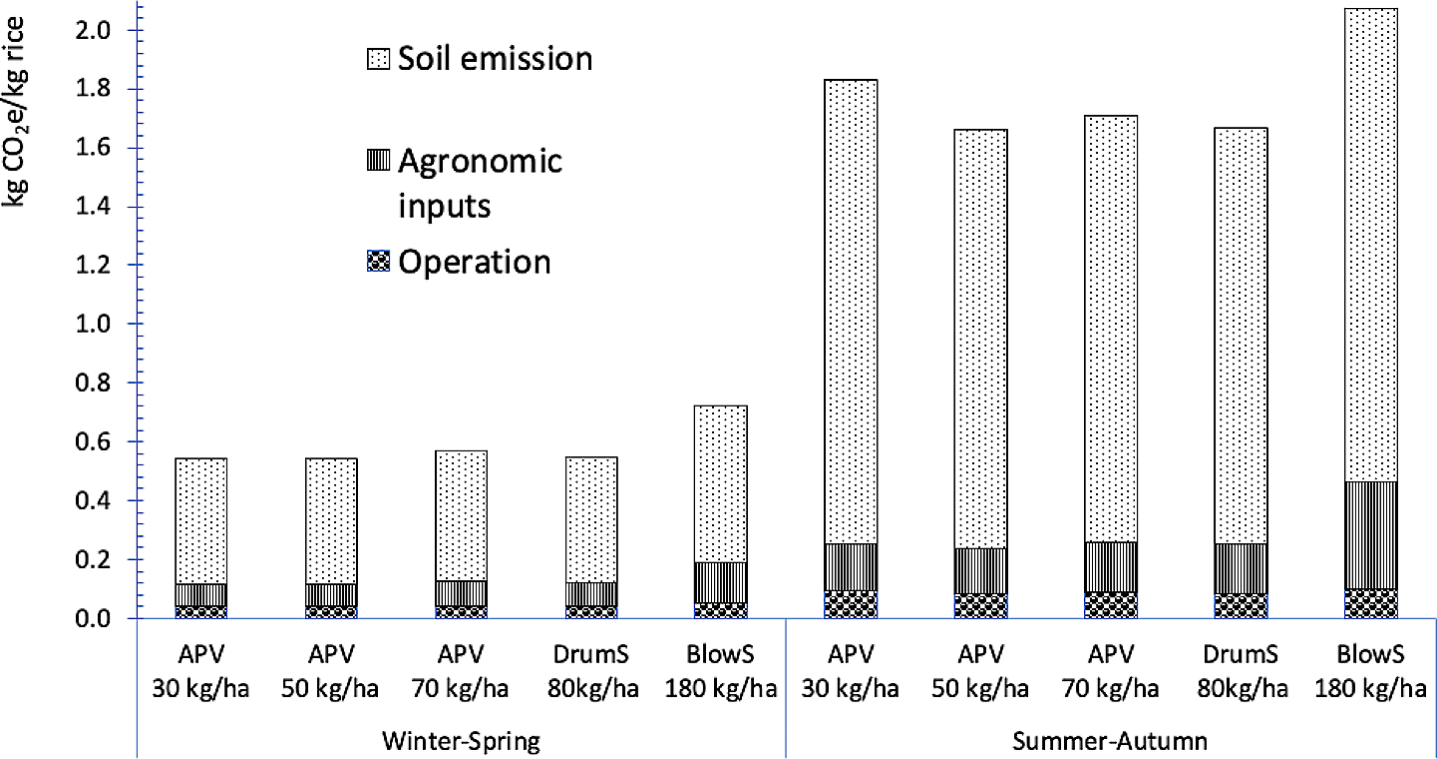
CF under different rice establishment methods during Winter-Spring 2020-21 and Summer-Autumn 2021

CF (GHGe per kg of paddy grains produced) was in the ranges of 0.54–0.73 and 1.67–2.07 kg CO_2_-eq kg rice^− 1^ in Winter-Spring and Summer-Autumn, respectively (Fig. 5). Because of the lower inputs, CF of mDSR was lower by 22–25% and 12–20% in Winter-Spring and Summer-Autumn, respectively, compared with bDSR. On the contrary, the highest CF was associated with bDSR, corresponding to its high agronomic inputs and lower yield. Of the total CF, agronomic inputs (excluding pesticide) contributed 9–19%, mechanized operations contributed 5–7%, and soil emissions contributed 74–86%.

## Discussion

Rice crop establishments in Mekong River Delta are mostly wet direct seeded rice in all cropping seasons; therefore, this study targeted identifying and developing the best DSR practice for only wet seeding. One of the trade-offs associated with the transition from transplanted to bDSR is the use of a high seed rate. The use of 180 kg ha^− 1^ seed rate is widely practiced in bDSR – the current most dominant method of rice establishment in Mekong River Delta. The experiment was conducted in a specific area and with a specific DSR machine. Out of the boundary and researched factors, other factors that can affect the analysis, such as soil conditions, equipment quality, technology, operation, radiation, wind direction, etc., were not considered. However, the experimental field was only 3.9 ha and located in one place in Can Tho province, so the said non-research factors would not strongly affect the comparison results.

**Fig. 6:**
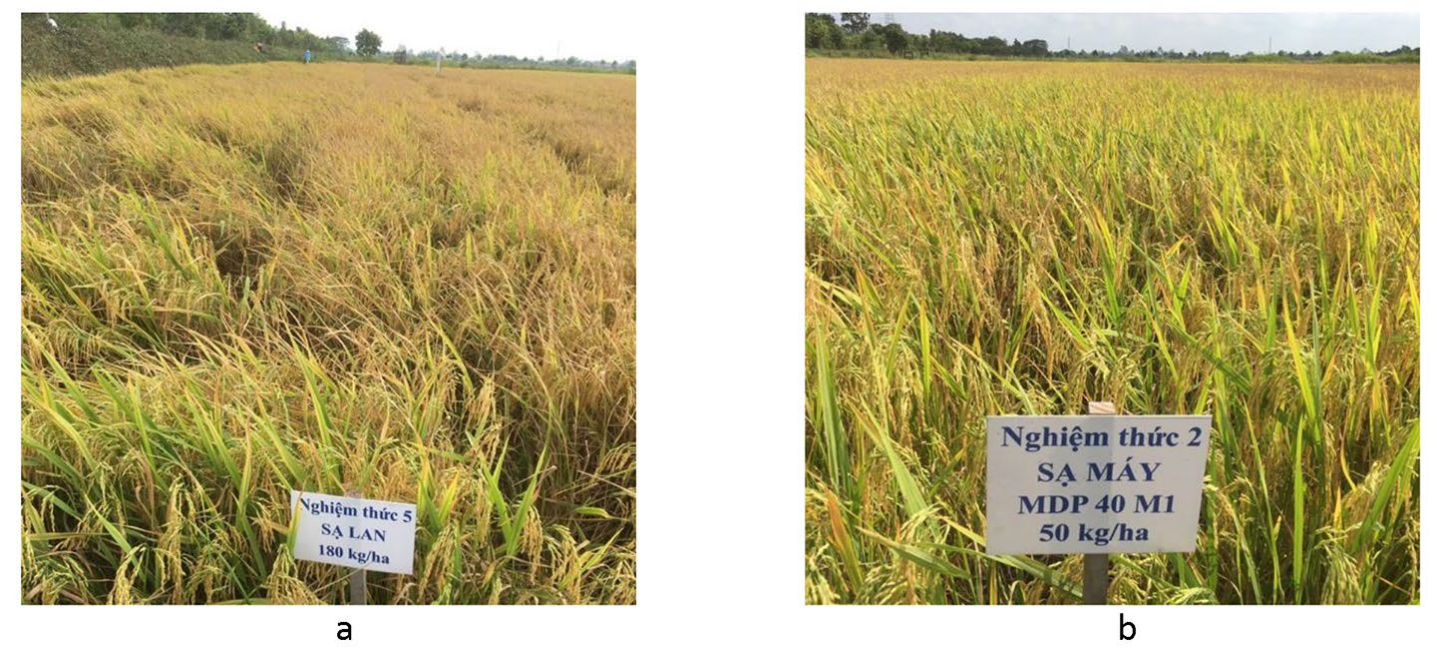
**a.** Broadcast seeding treatment. **b.** Mechanized direct seeding treatment

This study clearly illustrated the benefits of mDSR and dDSR over the current farmers’ practice of bDSR in terms of yield in Summer-Autumn season but more consistently in farm efficiencies, improving net income, and reducing the carbon footprint of rice production. Mechanized DSR can give a comparable advantage to that of mechanized transplanting (Nguyen-Van-Hung et al., 2022). The seedling uniformity of mDSR was significantly higher than that of bDSR. In addition, the precision crop establishment increased N-P-K use efficiency and productivity. In the case of mDSR-30, although it produced a lower yield than the mDSR-50, mDSR-70, and dDSR in Summer-Autumn, its N-P-K use efficiency was not significantly different from the said treatments because of its lower fertilizer use (*n* = 3, *p* < 0.05). In addition, mDSR also demonstrated another significant benefit of avoiding lodging risk leading to reduced postharvest losses (Fig. 6). similar to mechanized transplanting (Wang et al., 2014; Nguyen-Van-Hung et al., 2022). This could be one of the reasons for the higher yield of mDSR as compared to bDSR in some seasons.

Paddy grain yield in these study scenarios, 7–8 t ha^− 1^ in Winter-Spring season, are in agreement with that reported in the previous studies (Stuart et al., 2018; Nguyen-Van-Hung et al., 2022; Tho et al., 2021), but higher than that of most other countries in Southeast Asia (Statista, 2021). In contrast, broadcast-seeding rice cultivation requires high agronomic inputs (such as seeds, fertilizer, and pesticide), causing low income, as revealed in this study and recent scientific reports (Stuart et al., 2018; Devkota et al., 2020; Nguyen-Van-Hung et al., 2022). Therefore, with its demonstrated benefits, the mDSR-based rice production practice brings a good solution for farmers to reduce inputs and increase profitability, comparable to the One Must Do, Five Reductions practice – best agronomic package promoted by Ministry of Agriculture and Rural Development (MARD), Government of Vietnam in Mekong River Delta (Stuart et al., 2018; Nguyen-Van-Hung et al., 2022). Mechanized DSR is fully aligned with the One Must Do, Five Reductions practice program of the government as mDSR reduces seed rate (30–50 kg versus 180 kg ha^− 1^) which will enable farmers to afford certified seeds – one of the important recommendations of 1 M5R. As demonstrated, mDSR reduces seed rate and N rate – two of must reduction recommendations of One Must Do, Five Reductions practice. In addition, because of the low seed rate and line seeding, it is less prone to insect-pest and disease infestation and lodging, thereby potentially reducing pesticide usage and losses caused by pests and post-harvest losses associated with lodging. On the other hand, rice crop yield in Summer-Autumn is usually significantly lower than that of Winter-Spring (Stuart et al., 2018; Devkota et al., 2020), which is also the case for the current study. Furthermore, the grain yield generated from Summer-Autumn in the current study is significantly low because of the crop losses caused by pests, diseases, and flooding water status. It is also possible that the plants in Summer-Autumn were subjected to stress related to higher temperatures and lower solar radiations (Figs. 1 and 19–34 °C and 4–240 W m^− 2^ for Winter-Spring and 19–38 °C and 4–180 W m^− 2^ for Summer-Autumn), but the plants of mDSR were more vigorous and therefore were less sensitive to them. On the contrary, the Winter-Spring season has been observed as a rather favorable season/condition for rice growth and can hinder the treatment effects on grain yields. Based on our experience, lower seed rate with line seeding can yield higher or similar to high seed rate broadcast method - higher primarily in those season/year when there is more lodging or pest attack in high seed rate broadcast method as high seed rate broadcast method is more prone to lodging and pest attack. In season/year, less lodging and pest attack in high seed rate, yields are similar to low seed rate method. In line seeding, better aeration create less conducive to pest attack. Consequently, low-yield rice production resulted in low net income (13–261 $US ha^− 1^), the lowest for the bDSR requiring high inputs.

For the CF from rice production, aside from soil emission, the total CF also accounted for the additional components from agronomic inputs and mechanized operations. On the other hand, the CF of the researched scenarios was reduced from the application of alternate wetting and drying (water drainages during cropping season) and the removal of rice straw from the field. As the results, the total CF of Winter-Spring crop season (0.5–0.7 kg CO_2_-eq kg^− 1^ paddy grains) was comparable to the global range of CF reported by Wassmann et al. (2022) and lower by about 40% than that reported by Nguyen-Van-Hung et al. (2022) because of the reduced emission from rice straw removal. Meanwhile, CF in Winter-Spring (1.7–2.1 kg CO_2_-eq kg rice^− 1^) was more than 3 times higher than that of SA. The difference in CF between Winter-Spring and Summer-Autumn was mainly caused by the different methane emission factors applied in this analysis (1.7 and 2.8 kg CH_4_ ha^− 1^ day^− 1^ for Winter-Spring and Summer-Autumn, respectively) (Vo et al., 2017). It might be tempting to suggest that reducing the amount of seeds and fertilizers in bDSR would lead to increased yield and a reduction in CF, however, dDSR is a typical farmer practice in this region which often results in non-uniform crop establishment due to the non-uniform distribution of rice seeds. Reducing seed rates would have more missing plants and farmers need to have additional work to compensate for the gap through transplanting. Consequently, farmers prefer to have a higher seed rate and mDSR is a very good technique to have uniform crop establishment with less seed. On the other hand bDSR also has huge potential to reduce seed rate if it can be broadcast precisely. Drone based precision broadcast can reduce seed rate in broadcast method and this is new area of research we are working and will publish results soon.

Despite these benefits, adoption of mDSR is low because of the insufficient availability of machines and service providers. Scaling of mDSR can be more effective with integrated interventions, including business model development to increase access to capital-intensive technologies to smallholder farmers.

## Conclusion

This study introduced new technology and provided scientific evidence for the benefits of mDSR compared with the other direct seeded rice practices commonly applied for wet seeding in the Mekong River Delta of Vietnam, such as drum and broadcast seeding. mDSR significantly reduced seed rate and yield variability (vs. bDSR for this later), increased seeding precision, fertilizer use efficiency, and profitability, and reduced CF. mDSR applied seeds and fertilizer lower by 61–83% and 22–30%, respectively, but generated the same yield as bDSR. Consequently, mDSR increased N productivity 21–32% and 28–36% in Winter-Spring and Summer-Autumn, respectively, compared with bDSR. The differences in agronomic inputs also lead to better income from rice production applied mDSR. The net income of mDSR was comparable to that of dDSR but higher by 145–220 and 171–248 $US than that of bDSR in Winter-Spring and Summer-Autumn, respectively. In addition, the CF of mDSR was lower by 22–25% and 12–20% in Winter-Spring and Summer-Autumn, respectively, compared with bDSR. Given the above benefits of farming efficiency and low emission, mDSR would be a technology package that strongly supports sustainable rice cultivation transformation for Mekong River Delta of Vietnam.

## Electronic supplementary material

Below is the link to the electronic supplementary material.

Supplementary Material 1

## Nomenclature and units

$US: US dollar
bDSR: Blower broadcast seeding
CF: Carbon footprint (GHG emission per kg of product)
CO_2−_eq: Carbon dioxide equivalent
dDSR: Drum-seeding
GHGe: Greenhouse gas emissions
h: Hour
ha: Hectare
kg: Kilogram
kWh: Kilowatt*hour
K_2_O: Potassium
L: Liter
mDSR: Mechanized wet direct seeding
MC: Moisture content in a wet basis
N: Nitrogen
P_2_O_5_: Phosphorus pentoxide
t: Ton
